# 

**Published:** 1917-01-13

**Authors:** 


### CONTENTS.

PAGE

**The Problem of Repopulation** ... ... ... 293

**Compulsion for Medical Men?** ... ... ... 294

**Hospital and Institutional News** ... ... ... 295

Surgical Supplies Rejected by War Office—A Fine War Gift from New York—New Rules for Street Collections—Proposed War Hospital for Sunderland—The Charles Dilke Memorial Scheme—Where are the Hospital Chaplains?—The Voluntary System and Experiments on Patients—A Composite Medical Staff—A Dispute about Terms at a Red Cross Hospital—"Without Expense and Without Publicity"—Death of a Leicester Benefactor—The First Allocation of Miss Schaw's Bequests—Early Hospital Statistics for 1916—Panel Practitioners and Medical Mobilisation—Extensions at the Hertford County Hospital—The Rise in Railway Fares and Attendance at Rural Health Committees—Motor Ambulance Service in London—The Testing of Panel Dispensing—Another Link with Florence Nightingale and the Crimea—A Capital Comic Verse—The Artists and the Authors on War Service—Substitution of Women for Men in Insane Asylums—Allowances to Midwives—This Week's Drug Market—To Our Readers.

**Small-pox and War** ... ... ... ... ... ... 301

What Has Happened and may Happen Again.

PAGE

**Reform in Tuberculosis Work** ... ... ... 303

I. The Sanatorium.

**Medical Home-Truths.—I.** ... ... ... ... 304

**King Edward's Hospital Fund Statistics** ... 305

War's Effect on Relative Cost of London Hospitals.

**Hospital Architecture and Construction** ... 306

Maidenhead Cottage Hospital.

**The Matrons' and Sisters' Department** ... 307

Professional Registrationists and Others.

Round the Hospitals.

The Fell Beast and the Lady—The Much Maligned College—The R.B.N.A. and the Profession—The College and Irish Nurses—Farm Work during Holidays—Is there a Shortage of Nurses?

**The Heating of Hospitals** ... ... ... ... 309

XI. Pinewood Sanatorium.

**The Editor's Letter-Box** ... ... ... ... ... vi

Churches and Charity Funds.

**King Edward's Hospital Fund for London** ... vi

**The Institutional Worker** ... ... (Suppl.) 1

Precautions in Theatre Work at Operations.

Questions for January.

Enquiries and Answers.

The Sick and in Need.

Employment and Training.

